# Neurometabolic alterations in children and adolescents with functional neurological disorder

**DOI:** 10.1016/j.nicl.2023.103557

**Published:** 2023-12-21

**Authors:** Molly Charney, Sheryl Foster, Vishwa Shukla, Wufan Zhao, Sam H. Jiang, Kasia Kozlowska, Alexander Lin

**Affiliations:** a Department of Neurology, Columbia University Irving Medical Center, New York-Presbyterian, New York, NY, USA; b Center for Clinical Spectroscopy, Department of Radiology, Brigham and Women’s Hospital, Harvard Medical School, Boston, MA, USA; c Sydney School of Health Sciences, Faculty of Medicine and Health, The University of Sydney, Sydney, Australia; d Department of Radiology, Westmead Hospital, Westmead, NSW 2145, Australia; e Department of Psychological Medicine, The Children’s Hospital at Westmead, Westmead, NSW 2145, Australia; f Faculty of Medicine and Health, University of Sydney, Camperdown, NSW 2050, Australia; g Brain Dynamics Centre, Westmead Institute of Medical Research, Faculty of Medicine and Health, University of Sydney, Westmead, NSW 2145, Australia

**Keywords:** Functional neurological disorder, Functional seizures, Magnetic resonance spectroscopy, Excitatory/inhibitory neurotransmitters, MEGAPRESS, Neurometabolite

## Abstract

**Objectives:**

In vivo magnetic resonance spectroscopy (MRS) was used to investigate neurometabolic homeostasis in children with functional neurological disorder (FND) in three regions of interest: supplementary motor area (SMA), anterior default mode network (aDMN), and posterior default mode network (dDMN). Metabolites assessed included N-acetyl aspartate (NAA), a marker of neuron function; myo-inositol (mI), a glial-cell marker; choline (Cho), a membrane marker; glutamate plus glutamine (Glx), a marker of excitatory neurotransmission; γ-aminobutyric acid (GABA), a marker of inhibitor neurotransmission; and creatine (Cr), an energy marker. The relationship between excitatory (glutamate and glutamine) and inhibitory (GABA) neurotransmitter (E/I) balance was also examined.

**Methods:**

MRS data were acquired for 32 children with mixed FND (25 girls, 7 boys, aged 10.00 to 16.08 years) and 41 healthy controls of similar age using both short echo point-resolved spectroscopy (PRESS) and Mescher-Garwood point-resolved spectroscopy (MEGAPRESS) sequences in the three regions of interest.

**Results:**

In the SMA, children with FND had lower NAA/Cr, mI/Cr (trend level), and GABA/Cr ratios. In the aDMN, no group differences in metabolite ratios were found. In the pDMN, children with FND had lower NAA/Cr and mI/Cr (trend level) ratios. While no group differences in E/I balance were found (FND vs. controls), E/I balance in the aDMN was lower in children with functional seizures—a subgroup within the FND group. Pearson correlations found that increased arousal (indexed by higher heart rate) was associated with lower mI/Cr in the SMA and pDMN.

**Conclusions:**

Our findings of multiple differences in neurometabolites in children with FND suggest dysfunction on multiple levels of the biological system: the neuron (lower NAA), the glial cell (lower mI), and inhibitory neurotransmission (lower GABA), as well as dysfunction in energy regulation in the subgroup with functional seizures.

## 1 Introduction

Functional neurological disorder (FND) is characterized by a broad array of neurological symptoms—loss of motor function/paralysis, abnormal movements, functional seizures, and loss of sensory function—thought to reflect alterations within and between neural networks (Szaflarski and LaFrance, 2018, Diez et al., 2020, Perez et al., 2021, Rai et al., 2022). A recurring pattern of findings across studies is the increased activation of the brain’s emotion-processing regions—which include sub-regions involved in salience detection, arousal, self-referential processes, and emotion regulation—coupled with aberrant connectivity within the brain’s motor- and sensory-processing regions, potentially resulting in functional neurological symptoms (Perez et al., 2021, Pick et al., 2019). FND symptoms place substantial psychological, social, educational, and financial strains on children and their families, as well as a substantial burden on the health care system (Asadi-Pooya et al., 2021, Stephen et al., 2021). While FND symptoms resolve in most child and adolescent patients with treatment (Vassilopoulos et al., 2022), a small subset see their symptoms persist, leading to chronic impairment. In adult settings, the rate of chronic impairment is much higher. In this context, ongoing research is needed to broaden our understanding of FND and to aid in the development of novel treatments. Advanced magnetic resonance imaging (MRI) technologies—such as in vivo magnetic resonance spectroscopy (MRS)—allow for noninvasive assessment of neurometabolites. Metabolites assessed by MRS include N-acetyl aspartate (NAA), a marker of neuron function; myo-inositol (mI), a marker of glial cell function; choline (Cho), a membrane marker; glutamate plus glutamine (Glx), a marker of excitatory neurotransmission; γ-aminobutyric acid (GABA), a marker of inhibitory neurotransmission; and creatine (Cr), a marker of energy metabolism and of the global health of the underlying tissue (Maddock and Buonocore, 2012). MRS has been applied to many neurological and psychiatric disorders (Maddock and Buonocore, 2012, Oz et al., 2014).

No published studies have used MRS to study FND in children. In the adult FND literature, however, four studies using MRS have been reported. In ten patients with motor FND (n = 10), Demartini and colleagues (2019) measured NAA, Cr, Cho, mI, and Glx content in an emotion-processing region (anterior cingulate cortex [ACC]/medial prefrontal cortex [mPFC]) and in a motor-processing region (occipital cortex) (Demartini et al., 2019). Compared to healthy controls, patients with motor FND showed higher Glx/Cr in the ACC/mPFC but normal content in the occipital cortex. The higher Glx/Cr content in the ACC/mPFC correlated with alexithymia, anxiety, and severity of motor FND symptoms.

In 35 patients with functional seizures, Simani and colleagues (2020) measured NAA, Cr, Cho, mI, and Glx in dorsolateral prefrontal cortex (dlPFC), dorsomedial prefrontal cortex (dmPFC), ACC (regions involved in emotion processing), and thalami (regions involved in sensory and motor processing, and in regulating consciousness and alertness) (Simani et al., 2020). Compared to healthy controls, patients had lower NAA/Cr ratios in the dmPFC (bilaterally), right ACC, and thalamus (bilaterally). Patients also had lower Cho/Cr ratios in the right ACC and higher NAA/Cr ratios in the right dlPFC. Lower NAA/Cr ratios in the left thalamus and left DMPFC were associated with the frequency of functional seizures in the patient group. Lower NAA/Cr ratios in the right ACC and left PFC were also correlated with difficulties in attention and inhibitory control (assessed using Integrated Visual and Auditory Continuous Performance Test).

In a cohort of women with functional seizures (n = 20), Mermi and colleagues (2021) found that compared to healthy controls, NAA/Cho was reduced in the hippocampus, a region involved in learning and memory (Mermi et al., 2021). There were no differences in hippocampal NAA/Cr or Cho/Cr ratios.

And finally, in 24 patients with functional seizures, Mueller and colleagues (2023) combined MRI spectroscopy (measuring Cho/Cr, mI/Cr, and NAA/Cr) with brain temperature mapping (Mueller et al., 2023). In patients (compared to healthy controls) they found higher mI/Cr ratios in the precentral gyrus, posterior temporal gyrus, anterior cingulate gyrus, and orbitofrontal cortex. Patients (compared to healthy controls) also had higher brain temperature in the anterior cingulate gyrus, orbitofrontal cortex, and anterior cingulate gyrus, and lower brain temperature in the occipital cortex and frontal lobe. mI/Cr correlated with hostility, disability, and quality of life. There were no significant correlations with the serum inflammatory biomarkers.

Considered together, MRS studies suggest that adult patients with FND show changes in neurometabolic homeostasis across multiple regions: emotion-processing regions located in the prefrontal cortex; motor regions in the precentral gyrus; self-referential processes involving the posterior temporal gyrus; thalamic subcortical regions involved in sensory and motor processing, and in regulating consciousness and alertness; and the hippocampus, involved in memory and learning. All of these functional domains are known to be affected in patients with FND (Rai et al., 2022, Pick et al., 2019, Perez et al., 2021, Hallett et al., 2022).

While MRS has not been used to study FND in the pediatric population, other advanced imaging methods have been employed to uncover structural and functional differences in children with FND (Rai et al., 2022, Kozlowska et al., 2017, Kozlowska et al., 2018, Radmanesh et al., 2020). Most recently, a resting-state functional MRI study using independent component analysis (n = 31) identified wide-ranging connectivity changes in eight independent components corresponding to eight resting-state neural networks: language networks (IC6 and IC1), visual network, frontoparietal network, salience network, dorsal attention network, cerebellar network, and sensorimotor network (Rai et al., 2022).

The aim of the current study is to examine neurometabolic homeostasis in children with mixed FND—that is, various combinations of symptom presentations—compared to healthy controls, by quantifying the following neurometabolites: NAA, Cr, Cho, mI, Glx, and GABA. In addition, because children with FND present in a state of brain and body arousal (Rai et al., 2022, Radmanesh et al., 2020, Paredes-Echeverri et al., 2022)—arousal being greatest in children with functional seizures (Radmanesh et al., 2020)—we aimed to examine whether the children’s difficulties in regulating arousal was potentially reflected in alterations in the excitatory and inhibitory neurotransmitter balance (referred to as E/I ratio and determined by the ratio of Glx to GABA). Because data acquisition in MRS is time-consuming and children (including adolescents) are often unable to tolerate long periods in the MRI scanner, we chose three regions of interest based on the pediatric literature available at the time (Kozlowska et al., 2017, Kozlowska et al., 2018):

• SMA, a motor region that is part of the somatomotor network
• medial walls of the frontal lobes (ACC and medial PFC), which make up the aDMN
• posterior cingulate cortex (PCC) and precuneus, which make up the pDMN

We hypothesized that children with FND would show changes in neurometabolic homeostasis in all three regions of interest.

## 2 Methods

Thirty-two children admitted for treatment of FND to the inpatient Mind-Body Program at The Children’s Hospital at Westmead (Australia), during the period January 2019 to July 2021 agreed to participate in the current study. All children had undergone a comprehensive neurology assessment and had been given a DSM-5 diagnosis of FND by a pediatric neurologist (American Psychiatric Association, 2013). All had participated in a biopsychosocial assessment with the mind–body team: a structured interview with the child and family documenting the child’s developmental history, history of the presenting symptoms (including comorbid nonspecific symptoms), and functional disability rating on the Global Assessment of Functioning (GAF) scale. On self-report the children completed the Depression Anxiety and Stress Scales (DASS-21) and Early Life Stress Questionnaire (ELSQ) (see Table 1). On admission to the Mind-body Program resting-state HR was recorded.

**Table 1:** Summary of the measures used in the study.

**Measure**	**Description**
RAHC- GAF	The Royal Alexandra Hospital for Children Global Assessment of Function (RAHC-GAF) is the DSM-IV-TR GAF modified to include functional impairment secondary to physical illness (American Psychiatric Association, 2000). The scale has 100 points and 10 categories (10 points each). Healthy controls generally fall into the upper three brackets “superior in all areas” (score 91–––100), or “good in all areas” (score 81–––90). Lower values (and brackets) mark functional impairment of increasing severity. Patients with physical or psychological impairment fall into the lower brackets (score < 81).
DASS-21	The Depression Anxiety and Stress Scales (DASS-21)—total DASS score, but not the three subscales—are a validated measure of perceived distress in paediatric populations (Lovibond and Lovibond, 1995, Patrick et al., 2010).
ELSQ	The Early Life Stress Questionnaire (ELSQ) is a checklist of 19 stress items—and an option for elaboration—based on the Child Abuse and Trauma Scale (Cohen et al., 2006). Twelve items pertain to relational stressors, including the following: bullying; physical abuse; sexual abuse; emotional abuse; neglect; parental separation; loss by separation; loss by death; family conflict; severe illness of a family member; domestic violence; and other. Other items pertain to birth complications, life-threatening/severe illness, war trauma, and natural disasters. Participants record if they have or have not experienced the given stressor and the age period during which the stressor has been experienced.

Forty-one healthy controls were recruited from the same age bracket and geographical catchment area. Control participants were screened for the absence of mental health disorders, history of head injury, family history of mental health disorders, and chronic health concerns. All controls completed the DASS-21 and ELSQ, and were rated with the GAF (see Table 1).

The Sydney Children’s Hospital Network Ethics Committee approved the study. Participants and their legal guardians provided written informed consent.

### 2.1 MRI and MRS data acquisition

A Siemens PRISMA 3 T scanner (Siemens Healthineers, Erlangen, Germany, software version VE11C) together with a 64-channel head/neck phased-array RF coil was used for structural and MRS data acquisition. Two structural sequences were used for voxel placement. First, a 3D T1-W MP-RAGE was acquired sagittally with the following parameters and reformatted into three planes: TR = 2400 ms; TE = 2.21 ms; TI = 1150 ms; voxel size = 1.0 mm^3^ isotropic; iPAT = 3; FOV = 256 mm; matrix = 256 × 256; and acquisition time = 4 min 35 sec. Second, a 2D T2-W axial sequence was acquired with the following parameters: TR = 7490 ms; TE = 99 ms; slice thickness = 3 mm; iPAT = 2; FOV = 220 mm; matrix = 384 × 288; and acquisition time = 2 min 24 sec.

**Fig. 1:**
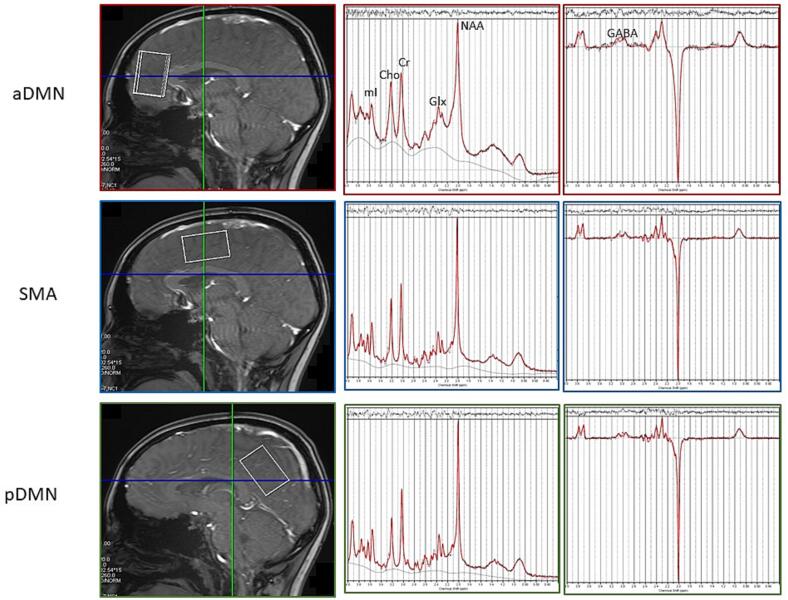
Representative PRESS and MEGAPRESS spectra from the aDMN (top), SMA (middle), and pDMN (bottom). Left: voxel location in T1-weighted sagittal image. Middle: Short echo PRESS (TE = 30 ms). Right: MEGAPRESS (TE = 68 ms). NAA: N-acetylaspartate, Glx: glutamate/glutamine, Cr: creatine, Cho: choline, mI: myoinositol, GABA: gamma amino butric acid.

MRS data were acquired using both point-resolved spectroscopy (PRESS) (Bottomley, 1984) and Mescher-Garwood point-resolved spectroscopy (MEGAPRESS) (Mescher et al., 1998) sequences in three locations; SMA (voxel size, 40 [AP] × 25 [RL] × 25 [CC] mm^3^), aDMN (voxel size, 25 [AP] × 30 [RL] × 35 [CC] mm^3^), and pDMN (voxel size, 25 [AP] × 25 [RL] × 40 [CC] mm ^3^) (Marjanska et al., 2013, Tremblay et al., 2014), as shown in Fig. 1. The MRS package was developed by Edward J. Auerbach and Małgorzata Marjańska and provided by the University of Minnesota under a C2P agreement (Marjanska et al., 2013, Tremblay et al., 2014). MEGAPRESS sequence parameters were as follows; TR = 2000 ms; TE = 68 ms; 64 averages (32 ON, 32 OFF); spectral width = 2000 Hz; editing pulse frequencies set to 1.9 ppm and 7.5 ppm for editing of GABA+; smf editing pulse bandwidth = 70 Hz. PRESS sequence parameters were as follows; TR = 2000 ms; TE = 30 ms; 32 averages, spectral width = 2000 Hz. Water-unsuppressed data were acquired for all voxel locations for both PRESS and MEGAPRESS acquisitions. Prior to data acquisition in each location, voxel-specific shimming was performed using the vendor-provided advanced-user 3D shimming process. Full width at half-maximum (FWHM) values were checked and recorded, and manual adjustments were made when line widths were greater than 14 Hz in the and SMA and pDMN and 18 Hz in the aDMN.

### 2.2 Data pre-processing

The single voxel MRS raw data were exported and pre-processed. The processing pipeline was implemented using OpenMRSLab and included channel combination using singular value decomposition (SVD), spectral registration to correct for frequency drifts, and residual water removal using the Hankel SVD method (Rowland et al., 2017). The metabolites were fit using linear combination models (LCModels) (Provencher, 1993). For the PRESS acquisition, total NAA (NAA + N-Acetyl aspartatyl glutamate), Cr (Cr + phosphocreatine), total Cho (tCho = phosphorylcholine + glycerophosphocholine), mI, and Glx (Glx = glutamate + glutamine) were quantified. For the MEGAPRESS acquisition, following channel combination, water subtraction and phase correction of the “on” and “off” spectra independently, the two spectra were subtracted in order to yield an edited spectrum. LCModels were then used to fit the edited spectrum and quantify GABA. Total creatine, quantified using the PRESS acquisition, was compared between FND and control groups in each region and then used to calculate ratios for all metabolites (NAA/Cr, Cho/Cr, mI/Cr, Glx/Cr, GSH/Cr, GABA/Cr). Prior studies of FND using MRS have employed creatine ratios (Demartini et al., 2019, Simani et al., 2020). The use of ratios in the study allows for more direct comparisons with the existing literature. In addition, the E/I ratio was calculated as PRESS Glx/MEGAPRESS GABA. Spectra were visually inspected and excluded if lipid peaks obscured the spectra such that it could not be reliably fit. Among PRESS acquisitions, spectra were included if the signal-to-noise ratio (SNR) > 20 and the Cramer-Rao lower bound (CRLB) for metabolites included in the analysis were CRLB < 10. For the MEGAPRESS acquisitions, spectra were included if the CRLB for GABA < 20. MRS acquisition and post-processing methods are summarized in Supplemental Table 1 (Lin et al., 2021).

### 2.3 Statistical analysis

Chi-square analyses and independent t-tests were used to calculate differences between the FND and control groups on categorical and continuous variables, respectively. Two-sided independent t-tests were used to determine whether creatine differed between the FND group and the control group.

Mixed linear effects models were implemented in R Version 3.6.1 to evaluate the effect of group (FND vs. control), to control for age and sex, and to account for associations among the metabolites for each region (R­CoreTeam. R, 2020). A separate model was created for each PRESS voxel acquisition (SMA, aDMN, pDMN). The use of mixed linear effects models decreases Type 1 error and does not assume that MRS metabolites in the same region are independent of each other. Apart from the use of mixed effects models, which decrease the number of models created, no other corrections for multiple comparisons were applied in the analysis. A symmetric correlation structure was used. Linear regressions with age and sex as covariates were used for GABA/Cr and E/I ratio in each of the three regions. The Shapiro-Wilks test was used to assess normality of all variables.

Pending clarification of Cr concentrations across the three regions of interest (FND vs. controls)—and in the scenario that no differences between groups were found—we planned to use metabolite ratios with Cr as a denominator, as was done in the four previous studies of FND in adults (Demartini et al., 2019, Simani et al., 2020, Mermi et al., 2021, Mueller et al., 2023).

The relationship between excitatory and inhibitory neurotransmitter balance—the E/I ratio—was examined using the ratio of glutamate and glutamine (excitatory neurotransmission) to GABA (inhibitory neurotransmission) in the three regions of interest. Spearman’s rho was used to examine the relationship between the E/I ratios in the aDMN and pDMN (the two key nodes of the default node network).

Within the FND group, associations between MRS metabolites (where group differences had been identified) and clinical measures (total DASS score, total ELSQ score, and resting-state heart rate) were evaluated using Pearson’s product-moment correlation coefficient or Spearman’s rho, depending on the normality of the involved variables.

Post hoc, within the FND group, independent two-sided student t-tests were used to compare neurochemical levels—and the E/I ratio—between FND participants who experienced functional seizures and FND participants who did not experience functional seizures. Significance was set to < 0.05 a priori.

Post hoc, group differences (FND vs. controls) were re-run following exclusion of the two participants with incidental findings. Post hoc, group differences (FND vs. controls) were also re-run with total DASS score—a composite measure of distress—included as a covariate.

## 3 Results

### 3.1 Participant characteristics

The final study groups for MRS acquisition comprised 32 children and adolescents (25 girls and 7 boys) with FND aged 10.00 to 16.08 years (mean = 13.33; SD = 1.48; median = 13.46) and 41 healthy controls (30 girls and 10 boys, aged 8.58 to 17.92 years [mean = 13.78; SD = 2.56; median = 14.33]). The groups were matched for sex (*χ*2 = 0.06; p = 0.80) and age (t(63.91) = −0.53; p = 0.596).

**Fig. 2:**
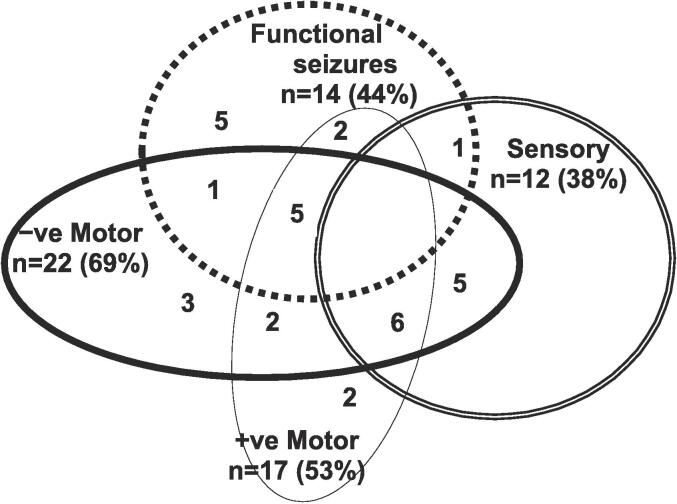
*Visual representation of functional neurological symptoms experienced by the children in the study cohort.* Children with mixed FND commonly present with multiple functional neurological symptoms. This figure depicts the functional neurological symptoms experienced by the 32 children with FND who were included in the analysis. Negative motor symptoms included: weakness or loss of function in the limbs, aphonia (loss of voice), and difficulties swallowing. Positive motor symptoms included: unusual gaits, difficulties with balance coupled with an uncoordinated gait, tics, tremors, dystonia, rumination (bringing up food via overactivation of the diaphragm), and dysphonia (change in the quality of the voice, e.g., a high-pitched baby voice). Sensory symptoms included: loss of touch, hearing, or vision. Functional seizures presented in a broad variety of ways and included faint-like events.

The clinical presentations of the 32 children with FND were diverse. They presented with one or more functional neurological symptoms (range, 1–8; mean = 3.47; median = 3.00) (Fig. 2). Length of illness ranged from 1 week to 12 months (mean = 4.27 months; median = 4.00 months), with over two-thirds (23/32; 72 %) having been ill for less than six months. Levels of functional disability at clinical assessment were high, with GAF scores ranging from 10 to 51 (mean = 32/100; median = 31/100) and days of school loss ranging from 0 to 20 weeks (mean = 5.33; median = 2.50) on presentation.

Twenty-eight children had had clinical medical imaging—MRI (n = 20), CT (n = 2), and MRI and CT (n = 6)—as part of their neurology workup (prior to their participation in the study and acquisition of the research MRS). Two children with FND had incidental MRI findings on clinical imaging: a cyst in the subcortical white matter of the right frontal lobe and a hyperintensity in the right thalamus. Both incidental findings remained unchanged over time with follow-up clinical imaging (3-year period). The other clinical imaging studies (26/28) had been reported as normal. Twenty children had had video EEGs which were also reported as normal. Clinical characteristics are reported in Table 2.

**Table 2:** Clinical and demographic information about participants with FND from clinical assessment.

**COMORBID MEDICAL CONDITIONS**		
Any comorbid medical condition	14	43.8 %
- Asthma/allergies	6	18.8 %
- Tourette’s syndrome	2	6.3 %
- Kidney disease	2	6.3 %
- Iron deficiency	2	6.3 %
- Epilepsy (a third was misdiagnosed with epilepsy)	2	6.3 %
- Hypothyroid	1	3.1 %
- Recurrent tendonitis	1	3.1 %
**COMORBID FUNCTIONAL SYNDROMES**		
Complex (functional) pain	26	81.3 %
- Lower limbs	13	40.6 %
- Headache	11	34.4 %
- Back/neck	9	28.1 %
- Abdomen	9	28.1 %
Any comorbid functional syndrome (excluding pain)	17	53.1 %
- Functional gut disorder	13	40.6 %
- Postural orthostatic tachycardia syndrome (POTS)	7	21.8 %
- Irritable bladder	1	3.1 %
**COMORBID NON-SPECIFIC SOMATIC SYMPTOMS**		
Any comorbid non-specific somatic symptom	30	93.8 %
- Sleep (difficulties falling asleep, waking, unrefreshing sleep)	27	84.4 %
- Concentration difficulties	24	75.0 %
- Dizziness	17	53.1 %
- Fatigue	14	43.8 %
- Nausea	14	43.8 %
- Heart palpitations (thumping heart)	13	40.6 %
- Breathlessness	9	28.1 %
- Sweatiness	8	25.0 %
**COMORBID MENTAL HEALTH DISORDERS AND SYMPTOMS**		
Any mental-health disorder (DSM-5)	29	90.6 %
- Anxiety disorder	27	84.4 %
- Depressive disorder	14	43.8 %
- ADHD	4	12.5 %
- PTSD	2	6.3 %
- Learning difficulties	2	6.3 %
- Autism	2	6.3 %
**MEDICATIONS AT THE TIME OF RESTING-STATE FUNCTIONAL MRI**		
Any medication at the time of testing	26	81.3 %
- melatonin for sleep	16	50.0 %
- SSRI for anxiety or depression	15	46.9 %
- small doses of second-generation antipsychotics for sleep	8	22.0 %
- clonidine for sleep or to down-regulate arousal	5	15.6 %
- methylphenidate for the treatment of ADHD	3	9.4 %
- medications pertaining to a functional gut disorder (e.g., antacids, anti-constipation medications, probiotics)	8	25.0 %
- replacements for deficiencies (thyroxine, iron, vitamin D)	5	15.6 %
- antiepileptic medications for epilepsy and in one case misdiagnosed epilepsy	3	9.4 %
- gabapentin for complex/chronic pain	2	6.3 %
- contraceptive pill	1	3.1 %
**COMMON ADVERSE CHILDHOOD EXPERIENCES (ACEs) REPORTED BY THE CHILD AND FAMILY**^a^**(maltreatment-related events are denoted by an asterisk)**		
One or more ACEs (range 1–10, mean 6, SD 2.5)	32	100 %
- Bullying by peers	20	62.5 %
- Child physical illness	19	59.4 %
- Family conflict	15	46.9 %
- Maternal mental illness	13	40.6 %
- Loss via separation from a loved one or a close friend	12	37.5 %
- Loss via death of a loved one	10	31.3 %
- Maternal physical illness	8	25.0 %
- Paternal mental illness	9	28.1 %
- Paternal physical illness	6	18.8 %
- Exposure to domestic violence*	6	18.8 %
- Emotional abuse (e.g., rejection/abandonment by a parent)*	6	18.8 %
- Sexual abuse*	4	12.5 %
- Physical abuse*	1	3.1 %
- Neglect*	1	3.1 %
**SOCIOECONOMIC STATUS OF THE FAMILY**		
Professional	9	28.1 %
White collar	10	31.3 %
Blue collar	11	34.4 %
Unemployed	2	6.3 %
**FAMILY CONSTELLATION**		
Biological parents	20	62.5 %
Blended family	10	31.3 %
Lives with one parent	2	6.3 %
**INTELLIGENCE QUOTA ESTIMATED FROM SCHOOL REPORTS (or formal testing)**		
Superior range (120 + )	8	25.0 %
Average range (80–––119)	18	56.3 %
Borderline range (70–––79)	5	15.6 %
Delayed (<70)	1	3.1 %

*denotes maltreatment events. Over a quarter of children (n = 10; 31.3 %) reported that they had experienced some form of maltreatment (including exposure to domestic violence).

a Bullying/social rejection by peers (n = 20) and illness (n = 19)—injury (n = 5); viral illness (n = 6); stress associated with managing a chronic medical illness (n = 5); severe allergic response (n = 2); and medical procedure (n = 1)—were the most common antecedent stressors, followed by parental mental illness (=16) and family conflict (n = 15).

Because of the high rate of comorbid functional, mental health, and medical conditions, many of the children (n = 26; 81.3 %) were on medication when admitted into the Mind-Body Program and when the MRS was acquired (see Table 2). Two controls were on maintenance medication for asthma; one was on the contraceptive pill; one was on a tetracycline antibiotic for acne; and one was on azathioprine for eczema.

Relative to controls, patients with FND had significantly higher total scores on the DASS (subjective distress) and lower scores on the GAF (see Table 3). On the ELSQ they reported more adverse childhood experiences (ACEs) across their lifespans (see Table 3).

**Table 3:** Comparisons between FND and healthy-control groups on Global Assessment of Function (GAF), Depression Anxiety and Stress Scales (DASS-21), Early Life Stress Questionnaire (ELSQ) and resting state heart rate.

**Measure**	**FND group (n = 32): mean value / total score** **(range)**	**Healthy-control group (n = 41): mean value/total score** **(range)**	**t /**χ^2^**(p)**
GAF	32.00 (10–51)	89.27 (75–99)	−30.45 (<0.001)
DASS-21 Total Score	24.22 (3–52)	5.54 0–30	6.96 (<0.001)
ELSQ	3.44 (0–9)	0.48 (0–3)	6.29 (<0.001)
HR (resting state; beats per minute)	89.37 (74–116)*	Not available	

*Resting state HR’s in the FND group were shifted to the right on the normative curve (Fleming et al., 2011). The lowest resting heart rate of 74 bpm in an adolescent aged 15.67 years sits on the 50th centile and the highest heart rate of 116 bpm in a child aged 14.58 years old sits on the 99th centile. In an earlier study of autonomic function in children with FND, the mean HR in controls—comparable to the current cohort in terms of sex and age—was 74.16 bpm (range 51.01––102.03), spanning values from the 3rd centile to the 95th centile (Kozlowska et al., 2015).

### 3.2 MRS quality measures

Across the three regions of interest and two acquisition techniques, 430 spectra were acquired (8 controls had missing data) (see Table 4). A total of 21 spectra were excluded, 9 because of their poor quality on visual inspection, 11 because of CRLB values inconsistent with inclusion criteria, and one because of an SNR < 20. Among the excluded spectra, 18/21 were in the aDMN. Descriptive statistics for measures of quality, SNR, full width half max (FWHM), and Cramer-Rao lower bound (CRLB) percentage for NAA, are listed in Table 5. Significantly poorer SNR (p = 0.01) was seen in FND patients compared to controls in the PRESS acquisition in the aDMN. SNR was subsequently included as a covariate in models of the aDMN PRESS metabolites. All other quality metrics for the PRESS acquisition were not significantly different between FND patients and controls. The CRLB percentage for GABA is reported for the SMA, aDMN, and pDMN MEGAPRESS acquisitions in Table 6. There are no significant differences in GABA CRLB percentage in any of the three regions between FND patients and healthy controls.

**Table 4:** Summary of missing data and spectra that were excluded from the analysis.

**Acquisition site**	**FND participant (n = 32)**	**Control participants (n = 41)**
**PRESS SMA**	32 spectra used in analysis	39 spectra used in analysis 2 missing data
**PRESS aDMN**	31 spectra used in analysis 1 excluded	35 spectra used in analysis 3 missing data 3 excluded
**PRESS pDMN**	32 spectra used in analysis	38 spectra used in analysis 3 missing data
**MP SMA**	31 spectra used in analysis 1 excluded	40 spectra used in analysis 1 excluded
**MP aDMN**	25 spectra used in analysis 7 excluded	34 spectra used in analysis 7 excluded
**MP pDMN**	31 spectra used in analysis 1 excluded	41 spectra used in analysis
**Total spectra used in analysis**	182	227
**Total missing data**	0	8
**Total excluded spectra**	10	11

**Table 5:** MRS PRESS Acquisition Quality Measures.

	FND				Controls			
PRESS	n	SNR Avg (SD)	FWHM Avg (SD)	tNAA %CRLB Avg (SD)	n	SNR Avg (SD)	FWHM Avg (SD)	tNAA %CRLB Avg (SD)
SMA	32	41.6 (2.8)	0.03 (0.005)	2.1 (0.2)	41	42.6 (2.8)	0.03 (0.007)	2.0 (0.2)
aDMN	31	34.7 (5.2)	0.06 (0.02)	2.8 (0.7)	38	37.8 (4.8)	0.05 (0.01)	2.8 (0.6)
pDMN	32	46.8 (1.6)	0.02 (0.003)	1.9 (0.3)	40	47.2 (2.2)	0.02 (0.003)	1.9 (0.3)

**Table 6:** MRS MEGAPRESS Acquisition Quality Measures.

	FND		Controls	
MP	n	GABA %CRLB Avg (SD)	n	GABA %CRLB Avg (SD)
SMA	31	5.3 (2.6)	40	4.8 (1.9)
aDMN	25	7.1 (4.0)	34	7.3 (4.4)
pDMN	31	5.4 (1.5)	41	6.3 (3.4)

### 3.3 MRS metabolites with water as a reference

Levels of creatinine—with water as a reference—were ascertained in the three regions of interest using PRESS (see Table 7). Creatine did not differ between the FND and control groups in any of the three regions of interest (p > 0.05): SMA t(41.56) = 1.71, p = 0.095; aDMN t(39.90) = 1.93, p = 0.061; and pDMN t(39.62) = 1.58, p = 0.123. Also documented in Table 7 are mean levels of NAA, mI, Cho, Glx from the PRESS analysis in FND and control participants across the three regions of interest.

**Table 7:** PRESS MRS Metabolites with reference to water in Children and Adolescents with FND and Healthy Controls.

	**tNAA**		**mI**		**tCho**		**Glx**		**Cr**	
**SMA**	Mean	SD	Mean	SD	Mean	SD	Mean	SD	Mean	SD
FND	6.97	0.48	3.58	0.26	1.14	0.09	8.26	0.39	4.25	0.22
Control	7.95	2.76	4.06	1.28	1.25	0.45	9.22	2.84	4.72	1.74

**aDMN**	Mean	SD	Mean	SD	Mean	SD	Mean	SD	Mean	SD

FND	5.11	0.77	2.79	0.51	1.00	0.13	7.67	1.20	3.55	0.44
Control	6.12	2.80	3.56	1.76	1.17	0.61	9.12	3.94	4.33	2.45

**pDMN**	Mean	SD	Mean	SD	Mean	SD	Mean	SD	Mean	SD

FND	7.68	0.29	3.61	0.24	0.93	0.08	8.85	0.44	4.50	0.16
Control	8.71	3.04	4.07	1.37	0.99	0.32	9.84	3.17	5.01	2.02

### 3.4 MRS metabolites with creatine as a reference

Descriptive statistics for metabolite ratios are presented in Table 8. Creatine did not differ between the FND and control groups in any of the three regions of interest (p > 0.05)(see previous section). In this context, in line with previous FND studies with adults, Cr was used as the denominator for metabolite ratios.

**Table 8:** MRS Metabolite Ratios in Children and Adolescents with FND and Healthy Controls.

	FND		Control		P-value
	Mean	SD	Mean	SD	
**SMA**	N = 32		N = 41		
tNAA/Cr	1.64	0.08	1.69	0.11	0.029*
GSH/Cr	0.30	0.03	0.30	0.02	0.923
mI/Cr	0.84	0.06	0.87	0.05	0.051
Glx/Cr	1.95	0.11	1.98	0.20	0.116
tCho/Cr	0.27	0.02	0.27	0.02	0.461
GABA/Cr	0.37‘	0.05	0.42^	0.07	0.009*
E/I	5.32‘	1.09	4.88^	0.95	0.094
E/I	4.91‘	1.03	5.13^	1.11	0.439
**aDMN**	N = 31		N = 38		
tNAA/Cr	1.44	0.15	1.45	0.15	0.896
GSH/Cr	0.20	0.07	0.20	0.06	0.081
mI/Cr	0.78	0.11	0.84	0.09	0.125
Glx/Cr	2.16	0.23	2.17	0.25	0.119
tCho/Cr	0.28	0.02	0.27	0.03	0.163
GABA/Cr	0.51**^#^**	0.16	0.55∼	0.29	0.688
E/I	4.75**^#^**	1.73	4.81∼	2.30	0.791
**pDMN**	N = 32		N = 40		
tNAA/Cr	1.71	0.09	1.76	0.11	0.038*
GSH/Cr	0.29	0.02	0.29	0.02	0.359
mI/Cr	0.80	0.06	0.82	0.05	0.069
Glx/Cr	1.97	0.12	2.00	0.19	0.108
tCho/Cr	0.21	0.01	0.20	0.02	0.284
GABA/Cr	0.41‘	0.06	0.41^	0.07	0.834
E/I	4.91‘	1.03	5.13^	1.11	0.439

Due to data quality control, there are some metabolic measures that were removed, these are indicated in the table as follows: ∼ n = 30, #n = 25,^n = 37, ‘n = 31. For p values, * and bold indicate significant difference.

**Fig. 3:**
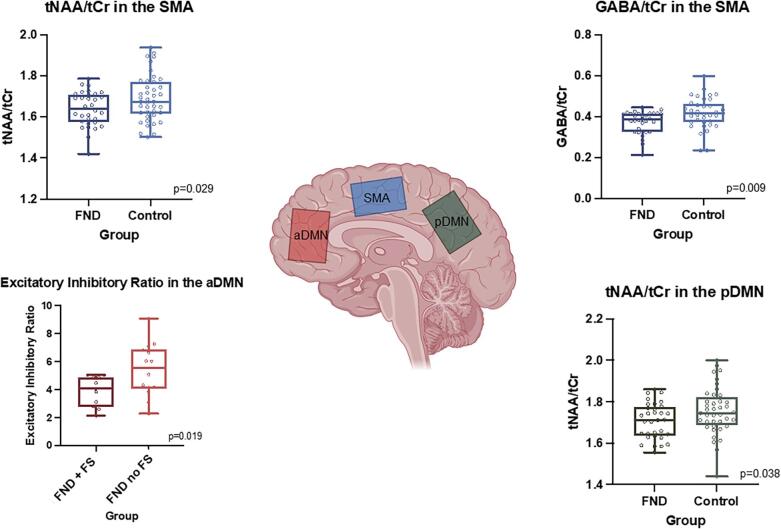
Significant Differences in Neurometabolites and E/I in the SMA, aDMN, and pDMN. Top left: N-acetylaspartate to creatine ratio (NAA/Cr) is reduced in patients with functional neurological disorder (FND) when compared to control in the SMA (p = 0.029). Top right: GABA is reduced in FND when compared to control in the SMA (p = 0.009). Bottom left: E/I ratio is reduced in FND patients with functional seizures (FND + FS) compared to those without (FND no FS). Bottom right: NAA/Cr is reduced in FND compared with control in the pDMN (p = 0.038).

In the SMA, participants with FND had lower NAA/Cr, mI/Cr (trend level), and GABA/Cr ratios, as shown in Fig. 3. In the aDMN there were no differences in metabolite ratios between groups (FND vs. controls). In the pDMN, participants with FND had lower NAA/Cr ratios and lower mI/Cr ratios (trend level). There were no differences in metabolites between FND participants with and without functional seizures.

Post-hoc analyses examined whether between group (FND vs. controls) findings held following exclusion of the two participants with incidental findings (a cyst in the subcortical white matter of the right frontal lobe and a hyperintensity in the right thalamus). The same pattern of findings was found. In the SMA, group differences were significant for tNAA/Cr, mI/Cr, and GABA/Cr. In the pDMN, group differences were significant for tNAA/Cr and mI/Cr.

Post-hoc analyses also examined whether findings pertaining to the effect of group (FND vs. control) held if total DASS score—a composite measure of distress—was added as a covariate. Decreased NAA/Cr in the SMA survived correction for total DASS score (p = 0.039). Decreased NAA/Cr in the pDMN survived correction for total DASS score (p = 0.043). Decreased GABA/Cr in the SMA and decreased mI/Cr (trend level) in the SMA and pDMN did not survive the correction for total DASS score.

### 3.5 Excitatory/inhibitory ratios

There were no significant differences between groups (FND vs. controls) for the E/I ratio in any of the three regions of interest. However, children experiencing functional seizures (n = 11) had lower E/I ratios in the aDMN compared to children with FND with no functional seizures (n = 14) (t(21.27) = −2.55; p = 0.018; mean = 3.90 vs. 5.42) and compared to controls (n = 33) (t(41.74) = −2.62; p = 0.012; mean = 4.00 vs. 5.53). Children with functional seizures (n = 14) also had lower E/I ratios in the pDMN (trend level) compared to healthy controls (n = 41) (t(52.79) = −2.00; p = 0.059; mean = 4.72 vs. 5.32).

There were no differences between the subgroup of children with functional seizures and the subgroup of children with other FND symptoms (but no functional seizures) on clinical measures: age (t(30) = 1.770; p = 0.087); sex (*χ*2 = 0.003; p = 0.957); total DASS score (t(30) = 0.268; p = 0.791); total ELSQ score (t(30) = 0.685; p = 0.499); GAF score (t(30) = −1.628; p = 0.114); total number FND symptoms (t(30) = −0.507; p = 0.616); total number of comorbid non-specific symptoms (t(18.81) = 0.726; p = 0.477); acute vs. chronic illness presentation (*χ*2 = 0.003; p = 0.960); and presence/absence of a comorbid mental health disorder (*χ*2 = 0.146; p = 0.702).

### 3.6 Correlational analyses with clinical measures

Within the FND group, there was a negative correlation between resting-state heart rate and SMA mI/Cr (r(30) = − 0.467, p = 0.007) and resting-state heart rate and pDMN mI/Cr (r(30) = − 0.480, p = 0.005). In other words, increased arousal (indexed by higher heart rate) was associated with lower mI/Cr in the SMA and pDMN. There was a positive correlation between total ELSQ score and SMA tNAA/Cr (r(31) = 0.389, p = 0.028). The Bonferroni corrected p value was 0.007.

## 4 Discussion

The current study is the first to examine neurometabolic homeostasis in children with mixed FND compared to healthy controls. We measured several neurometabolites—NAA, mI, Cho, Glx, GABA, and Cr—in the SMA, aDMN, and pDMN. Children with FND had lower NAA/Cr, mI/Cr (trend level), and GABA/Cr ratios in the SMA. Children with FND also had lower NAA/Cr and mI/Cr (trend level) ratios in the pDMN. Pearson correlations found that increased arousal (indexed by higher heart rate) was associated with lower mI/Cr in the SMA and pDMN. Analyses pertaining to excitatory and inhibitory neurotransmitter balance—indexed by the E/I ratio—showed that children with functional seizures had lower E/I ratios in the aDMN compared both to children without functional seizures and to healthy controls, and lower E/I ratios in the pDMN (trend level) compared to healthy controls. Post-hoc analyses—using total DASS score as a covariate—suggested that subjective distress modulated changes in GABA/Cr (in the SMA) and changes in mI/Cr (in the SMA and pDMN). The potential implications of our findings are discussed below.

### 4.1 N-acetyl aspartate (NAA)

NAA is the largest peak identified by MRS. NAA—a precursor to enzyme synthesis, and involved in neuronal osmoregulation, and axon–glial signaling—is synthesized in the mitochondria of neurons and is present in the cell body, axons, and dendrites (Moffett et al., 2007). It is considered a marker of neuronal health, viability, number of neurons, and functional capacity of neuronal mitochondria (Maddock and Buonocore, 2012). Since children with FND are expected to achieve a full recovery with treatment (Vassilopoulos et al., 2022), the decreased NAA in the SMA and pDMN may potentially reflect reversible neuronal or mitochondrial dysfunction. Alternately, it may be a byproduct of glial dysfunction due to altered glial metabolism or dysregulated neuronal–glial signaling. A previous study of adults with FND similarly observed lower NAA/Cr ratios in various brain regions, including the dmPFC, right ACC, and thalamus (Simani et al., 2020).

The SMA lies in the dmPFC (superior frontal gyrus) and is involved in motor planning, motor control (initiation and inhibition), error detection, decision uncertainty, feedback regarding unfavorable outcomes, and subjective urge to move (Fried et al., 1991, Nachev et al., 2008, Haggard, 2008, Zhang et al., 2012). These functions are processed along an anterior–posterior continuum. The anterior part of the SMA (pre-SMA) is more involved in complex situations with cognitive- or emotion-related inputs. The posterior part of the SMA (SMA-proper) is more involved in action. The entire complex is part of the cortico-striato-thalamo-cortical circuit system. The pre-SMA has strong connections with emotion-processing regions (epithalamus and orbitofrontal cortex) and decision-making regions (dlPFC). The SMA-proper has strong connections with the primary motor cortex (Nachev et al., 2008, Haggard, 2008, Zhang et al., 2012). Because of the broad range of functions mediated by the SMA, the decreased NAA/Cr ratio in the SMA in children with FND, coupled with decreased mI/Cr (trend level) and GABA/Cr ratios, presumably contributes to symptoms across motor-sensory, emotion-processing, and cognitive-control domains. In the current study children with FND had symptoms across all three domains: motor-sensory (see Fig. 1), emotion processing (see Table 4 reporting DASS score for subjective distress and autonomic arousal indexed by resting-state heart rate), and cognitive control (see Table 2 reporting subjective difficulties in concentration in 75 % of the sample). On a broader level, the above findings are consistent with Yeo’s observation that “sensory and motor areas are embedded within cerebral networks” (p 1150) (Yeo et al., 2011), with the consequence that functional changes in the SMA have important flow-on effects across multiple neural networks.

In the current study, the pDMN region of interest included the precuneus and the PCC. Changes in connectivity involving the precuneus are implicated in adult FND studies. A pediatric EEG study using low-resolution brain electromagnetic tomography (LORETA) localized neural sources to the dorsal anterior cingulate cortex/dmPFC, mid cingulate cortex, PCC/precuneus, and SMA (Kozlowska et al., 2018). The precuneus and PCC are involved in emotion regulation, self-agency, self-referential processing, and memory recall (episodic memories and recall of memories are better when they are related to the self) (Loeffler et al., 2018, Zhao et al., 2018). Akin to their adult counterparts, children with FND show a loss of self-agency in relation to their FND symptoms: the symptoms are experienced as involuntary. Lower NAA/Cr ratios in the pDMN, coupled with decreased mI/Cr ratios (trend level), may potentially contribute to this loss of self-agency.

### 4.2 Myo-inositol (mI)

Lower mI/Cr ratios (trend level) were identified in the SMA and pDMN of children with FND (vs. controls). These differences were no longer apparent when total DASS score was added as a covariate, suggesting that subjective distress presenting as depression, anxiety, and the subjective and physiological experience of being stressed, modulated mI/Cr ratios to come degree. Along the same lines, correlation analyses found an association between increased arousal (indexed by higher heart rate) and lower mI/Cr ratios in the SMA and pDMN, suggesting a close relationship between mI/Cr and level of autonomic system arousal.

Myo-inositol is a simple isomer of glucose. It is found in higher concentrations in glial cells (Kim et al., 2005, Haris et al., 2011), the brain’s immune-inflammatory cells (Frank et al., 2007), and is therefore considered a marker of glial cell function. Notwithstanding, mI is also found in neurons, where levels (higher than those in plasma) are maintained by a sodium mI transporter (Fisher et al., 2002).

mI is a precursor of membrane phospholipids. In this role it is involved in osmoregulatory processes and in cell membrane and myelin sheath structures (Haris et al., 2011). mI is also a precursor of phosphoinositides. In this role it is involved in second messenger systems: the phosphatidylinositol (PI-cycle) signal transduction pathways in the brain (Kim et al., 2005, Zulfarina et al., 2019). Signal transduction pathways form complex signaling networks that allow neurons to receive, process, and respond to information (Kim et al., 2005, Bhalla and Iyengar, 1999). Given the diversity of the receptors coupled to the PI-cycle, alterations in mI concentration—and any resultant perturbation in PI-cycle functioning—may affect neural network function through changes in receptor density, modulation of downstream cellular events, and other forms of epigenetic expression (Kim et al., 2005).

Glial cells are heterogeneously distributed throughout the brain (Mittelbronn et al., 2001). In contemporary neuroscience, glial cells are conceptualized as key homeostatic regulators of the central nervous system—guardians of neuronal excitability (Verhoog et al., 2020), neuroplasticity (Um, 2017), and connectivity. In illness states, they can drive abnormal neuronal activity (Robel and Sontheimer, 2016), illness-promoting neuroplasticity processes, and aberrant neural network function (Um, 2017, Lee and Chung, 2019).

Glial cells may also serve as immunosensors of the stress response (Frank et al., 2019, Frank et al., 2020). They “display an amazing repertoire of functions that put them in the unique position to sense and respond rapidly to alterations in homeostasis and integrate the neural response to threat” (p183) (Frank et al., 2019). This role implicates them as key players in the neurobiology of stress-related disorders, including FND (Kozlowska et al., 2018, Frank et al., 2019, Frank et al., 2020, Stephenson and Baguley, 2018).

In the current study, children with FND reported increased adverse childhood experiences across their life spans and also reported that stress events (physical, psychological, or mixed) typically triggered their clinical presentations (see Table 3, Table 4). Children with FND also showed elevated scores of depression, anxiety, and stress, on the DASS. And their resting-state heart rates were shifted to the right on the normative curve reflecting increased autonomic arousal (see Table 3) (Fleming et al., 2011). If glial cells play a role as immunosensors of the stress response, stress-induced priming of glial cells—via recurrent activation due to physical or psychological stress (and associated increases in arousal)—may play an important role in triggering the FND illness (Frank et al., 2019, Frank et al., 2020, Frank et al., 2016). Stress-induced priming reflects a state of glial cell readiness that occurs because of previous exposure to stress hormones (such as glucocorticoids and catecholamines). The primed glial cell holds memory for past stress and activates in a potentiated manner in response to subsequent stress.

An additional hypothesized mechanism suggests that glial cells primed by stress may result in a breakdown of glial-medicated synapse-modification processes, thereby enabling the illness to be maintained over a period of time or, in a subset of patients, to become chronic (Stephenson and Baguley, 2018).

Finally, we note that biological-marker research in children with functional seizures shows a neurophysiological state of increased autonomic and motor-respiratory system activation analogous to that seen during episodes of panic in patients with panic disorder (Kozlowska et al., 2017, Sawchuk et al., 2020). Along the same lines, in a study of pediatric patients with functional seizures, it was found that approximately 50 % of participants triggered their functional seizures by hyperventilating (also a feature of panic disorder). Hyperventilation temporarily destabilizes the child’s acid-base balance—inducing a respiratory alkalosis (secondary to low arterial CO2) (Kozlowska et al., 2017)—and functions (initially) to increase excitability within neural networks (Sparing et al., 1985, Jensen et al., 2002, Carbon et al., 2000, Stenkamp et al., 2001). It has been hypothesized that these physiological changes (increased brain and body arousal) increase the likelihood of neural network instability and dysregulation, which then manifest in the emergence of a functional seizure (Radmanesh et al., 2020, Kozlowska et al., 2017). In patients with panic disorder, hyperventilation also leads to excessive production of lactate in glial cells and to an accumulation of lactate in the extracellular fluid (Maddock and Buonocore, 2012). Lactate accumulation shifts PH in the direction of acidosis, functions as a buffer to the respiratory alkalosis, and may, potentially, trigger increased intake of mI at the PH-sensitive sodium mI transporter (found on both glial cells and neurons^2^) (Di Daniel et al., 2009) (Fisher et al., 2002, Uldry et al., 2001). Our findings of decreased mI/Cr (SMA and pDMN) in children with mixed FND and Mueller and colleagues (2023) (Mueller et al., 2023) findings of increased mI/Cr ratio (precentral gyrus, posterior temporal gyrus, anterior cingulate gyrus, and orbitofrontal cortex) in adults with functional seizures, suggest potential involvement (dysregulation) of the acid-base system. Accordingly, both lactate and MI may be involved in altered acid-base regulation that underpins—at least in part—altered function of acid-sensitive fear circuits (Maddock et al., 2013). While formal resting-state spectroscopy assessments of brain mI levels in panic disorder have yet to be conducted (Ham et al., 2007),^3^ two small, randomized control trials point to the therapeutic benefit of mI supplementation for the management of treatment-resistant panic disorder (Zulfarina et al., 2019, Benjamin et al., 1995, Palatnik et al., 2001, Concerto et al., 2023). This emergent literature raises the intriguing possibility that mI supplementation could potentially be useful in the treatment of FND, and in particular the subgroup of patients with functional seizures with concurrent hyperventilation or comorbid panic disorder. In this subgroup, mI supplementation could be trialed—using a randomized approach—together with the psychological interventions that are part of current biopsychosocial interventions (Savage et al., 2022, Kozlowska et al., 2023, Fobian et al., 2020, Sawchuk et al., 2020). A summary of MRS studies in panic disorder is available in Maddock and Buonocore, 2012, Maddock et al., 2013 (Maddock and Buonocore, 2012, Maddock et al., 2013).

In sum, our findings of lower mI/Cr ratios (trend level) in the SMA and pDMN and our findings of arousal/subjective distress as potential modulators of mI/Cr levels, coupled with increasing understanding about the role of glial cells in neural network function, suggest that glial cells and mI-based functions may play an important role in both triggering and maintaining the aberrant changes in metabolic activity and neural network function that underpin FND symptoms in children (Rai et al., 2022).

### 4.3 γ-aminobutyric acid (GABA)

GABA has not been previously quantified in patients with FND. GABA, an amino acid, is the primary inhibitory neurotransmitter for the central nervous system: it reduces neuronal excitability by inhibiting nerve transmission (Allen et al., 2022). GABAergic neurons are most prominent in the hippocampus, thalamus, basal ganglia, hypothalamus, and brain stem. In the SMA, local GABAergic interneurons are thought to contribute to motor control by gating incoming inputs (Yang et al., 2021, Estebanez et al., 2017). Well-functioning GABA-mediated processes maintain cortical excitability—which is increased in children with FND (Radmanesh et al., 2020, Kozlowska et al., 2017)—within normative parameters. For example, high-frequency gamma band oscillations “depend on the rhythmic activity of local networks of GABAergic interneurons via their synchronizing effects on the output of glutamatergic excitatory neurons” (p 216) (Maddock and Buonocore, 2012, Mann and Mody, 2010). In children with FND (versus healthy controls) high-frequency oscillations are increased in the resting state (Radmanesh et al., 2020) and in response to voluntary hyperventilation (which functions to increase cortical arousal) (Braun et al., 2021).

Importantly, glial cells contribute to inhibition processes in many ways. Glial cells engage in reciprocal signaling with neurons—also using GABA as a neurotransmitter—and they modify neuron inhibitory and excitatory synapses (Um, 2017, Angulo et al., 2008, Velez-Fort et al., 2012).

In the previous section we noted the conceptual overlap between pediatric FND, hyperventilation, and panic disorder. It is therefore of interest to know that lower levels of GABA—on resting-state MRS—is a recurrent finding in adults patients with panic disorder (Ham et al., 2007, Goddard et al., 2001, Long et al., 2013). Also of interest is a recent study suggesting that decreased GABA levels in the anterior and posterior cingulate, may contribute to difficulties in cognitive function in patients with mild cognitive impairment (Fu et al., 2023).

In this context, our finding of decreased GABA/Cr in the SMA suggest a decreased capacity for inhibition, but the finding is not specific at to which aspect of this complex system—motor control, emotion-process, or cognitive control—is dysregulated.

### 4.4 Excitatory and inhibitory neurotransmitter balance

Allostasis “is the process by which the brain efficiently maintains energy regulation in the body” (p 1) (Kleckner et al., 2017). The energy-regulation process is thought to involve ascending interoceptive pathways, brain regions important for interoception, and a distributed intrinsic allostatic–interoceptive system in the brain that manages energy regulation in a predictive manner, in which “the brain continually anticipates the body’s energy needs in an efficient manner and prepares to meet those needs before they arise” (p 1) (Kleckner et al., 2017, Craig et al., 2010, Craig, 2013, Jungilligens et al., 2022). The distributed allostatic–interoceptive system is thought to comprises the salience and the default mode networks (Kleckner et al., 2017). One can hypothesize that dysregulation within either of these two networks or within any of their components is likely to affect energy regulation and also regulation of other emotion-processing functions. This issue is of particular interest in FND because multiple studies have shown that children with FND—and children with functional seizures, in particular—are characterized by a state of increased resting-state arousal (Radmanesh et al., 2020, Paredes-Echeverri et al., 2022). Moreover, children with functional seizures demonstrate difficulties in regulating their biological systems back to a healthy baseline state following exposure to a stressor (Kozlowska et al., 2017, Braun et al., 2021). In the adult literature, two intriguing studies used brain temperature mapping to show elevations in brain temperature across multiple brain regions in patients (primarily women) with functional seizures (Mueller et al., 2023, Sharma and Szaflarski, 2021). The authors interpreted these findings as reflecting stress-induced neuroinflammation in the context of adverse childhood experiences. On the cellular/neurometabolites system level, the finding could reflect aberrant activation of glial cells (as immunosensors of the stress response; see previous subsection); loss of biological coherence and inefficient utilization of energy resources (reflecting as stress-related adaptation, also known as allostatic load); or a combination of both processes.

In the current study, we assessed energy regulation using the construct of excitatory-inhibitory (E/I) balance, indexed by the E/I ratio. E/I balance is essential for cell membrane stability and flow of information through the neural network (Selten et al., 2018). Both chronic and acute stress can affect E/I balance, and the direction of change varies in different studies (Han et al., 2020). Within the FND group, children with FND with functional seizures had lower E/I ratios in the aDMN (vs. those with FND without functional seizures and vs. controls) and pDMN E/I (vs. controls, trend level). Because flow of information through the neural network requires energy, alterations in E/I balance in children with functional seizures may reflect difficulties in energy regulation and energy flow.

### 4.5 Modulation of neural metabolite changes by subjective distress (total DASS score) and arousal

We found that group differences (FND vs. controls) were no longer apparent for SMA GABA/Cr, SMA mI/Cr (trend level), and pDMN mI/Cr (trend level) when Total Dass Score—a measure of subjective distress—was added as a covariate. These findings suggest that subjective distress modulated these neural metabolites to some degree. Moreover, within the FND group, negative Pearson’s correlations between heart rate and mI/Cr in the SMA and pDMN suggest that physiological arousal (reflecting decreased restorative vagal activity and increased sympathetic activity) was involved in modulation of mI/Cr in these regions. Considered together, these data hint at complex biological processes involving the stress system (including psychological processes and brain-body arousal systems) that may allow adverse life experiences to be biologically embedded and come to be expressed in altered neural metabolite levels and altered neural network connectivity patterns (Rai et al., 2022).

### 4.6 Potential role of medications

The patients in this study were sufficiently unwell to necessitate admission to hospital and the use pharmacotherapy as part of the inpatient treatment intervention (see Table 2). The most common medications included melatonin for sleep (50 %), a selective serotonin reuptake inhibitor (SSRI) for anxiety or depression (46.9 %), a small dose of quetiapine, a new generation antipsychotic medication to help with sleep (22 %), and use of clonidine to down-regulate arousal (15.6 %) (see Table 2). The potential effects on neurometabolites are briefly noted.

Melatonin (5-methoxy-N-acetyltryptamine) is neuroprotective. Melatonin is involved in anti-oxidant, anti-inflammatory, and immune-regulation functions (Acunacastroviejo et al., 1995, Reiter et al., 2000, Ahmad et al., 2023). In addition, melatonin and clonidine have been shown to down-regulate arousal by enhancing GABA-receptor activity or GABA content in the brain (Yu et al., 2023, Czyzewskaszafran et al., 1991). In this context, our finding of lower GABA/Cr in the SMA is unlikely to be related to pharmacotherapy with melatonin or clonidine (the findings being in the opposite direction from the neuroprotective and GABA-enhancing roles of these medications). Along the same lines, however, it is possible that the neuroprotective/anti-arousal effects of melatonin and clonidine may have attenuated/masked the full extent of GaBA/Cr decreases in the SMA or may have masked any potential GABA/Cr decreases in the aDMN and pDMN.

A small literature suggests that depressed adult females show attenuated Cho/Cr, that SSRIs help to increase the Cho/Cr ratio in the direction of normal (Zhang et al., 2015) and that, in the rat prefrontal cortex, quetiapine increases glutamate levels (with no effect on GABA) (Yamamura et al., 2009). In the current study, however, there were no differences in Cho/Cr and glutamate/Cr ratios between participants with FND and healthy controls, and the effects of SSRIs and quetiapine are difficult to determine.

### 4.7 Limitations of this study

There are several limitations to this study. First, the generalizability of our findings is limited by the small sample size. Notwithstanding, compared to previous studies of MRS in patients with FND (Demartini et al., 2019, Simani et al., 2020, Mermi et al., 2021); our sample size is larger or of a similar size. Our sample size was further limited in the aDMN due to the exclusion of spectra during quality assurance. The aDMN voxel is located close to the skull, leading to increased risk of lipid contamination of the spectra. It is more difficult and more error-prone to quantify the neurometabolites of interest from spectra with lipid contamination. Because the aDMN was the area with the most data excluded, the E/I imbalance measures between the two subgroups of patients, should be considered with caution. Second, our pediatric FND cohort was characterized by a mixed FND symptoms in different combinations. While this clinical picture is typical of pediatric patients and provides generalizable evidence of neurometabolite changes in children with FND, it makes the findings less specific to any particular subtype of FND. Third, this is a cross-sectional study. It precludes any determinations of causality and does not provide information about whether the neurometabolite changes identified in this study normalize on resolution of the illness. Fourth, because the task of lying still in an MRI scanner is challenging for children, we limited out study to three areas of interest. Future pediatric studies will need to both replicate our finding and to examine other areas of interest: the amygdala, cerebellum, temporo-parietal junction, and nuclei within the basal ganglia (Blakemore et al., 2016, Espay et al., 2018, Roelofs et al., 2019). Fifth, because children with functional seizures show persisting activation of high-frequency (beta power) bands following hyperventilation (Braun et al., 2021), and because hyperventilation triggers functional seizures in a substantial proportion of cases (Kozlowska et al., 2017, Sawchuk et al., 2020), future studies should use functional MRS to look at neurometabolites in response to a metabolic or emotional challenge (e.g., hyperventilation, or a visual task designed to evoke an emotional response). Functional MRS studies in patients with panic disorder have shown an elevated lactate response to metabolic challenges (Maddock and Buonocore, 2012) and a diminished Glx response that is temporally decoupled from lactate responses, reflecting a temporary metabolic dysregulation and pH dysregulation associated with altered function of acid-sensitive fear circuits. Whether such changes also contribute to the neural network dysregulation seen in functional seizures (Rai et al., 2022, Madec et al., 2020)—and in particular the subgroup of such patients who trigger their functional seizures by hyperventilation—is of substantial clinical interest. Theoretically, a buildup of lactate (which makes the extracellular fluid more acidic) would also intersect with mI transportation into neurons since the Na + mI transporter is pH dependent (with increased transportation as pH decreases [becomes more acidic]). mI supplementation, as noted above, is used in the treatment-resistant panic disorder. In this context, further work to examine the neurometabolite changes in FND, both in the resting state and in response to metabolic challenges, would clarify the potential overlap with panic disorder and the potential for alternate treatments. Finally, future studies with larger sample sizes should focus on the longitudinal application of multimodal imaging to characterize how imaging measures are associated with resolution of the FND illness in children. If large enough, such studies may also be able stratify by clinical presentation.

## 5 Conclusions

Our findings of multiple differences in neurometabolites in children with FND compared to healthy children suggests dysfunction on multiple levels of the biological system—neurons, glial cells, and neurotransmitters/signaling. All presumably combine to contribute to symptoms experienced by our child and adolescent patients with FND across motor-sensory, emotion-processing, and cognitive-control domains. The potentially important role of glial cells in mediating neurometabolite changes—via glia cell signaling and intercellular signaling in neuron–glia networks—suggests a need for a broader research lens, one that conceptualizes glial cells as key players within neuron–glia networks. This broader lens could open the door to further research and new treatment options in the future. Our findings pertaining to the potential role of arousal and subjective distress in modulating neurometabolites changes, cohere with the idea that FND is a disorder that emerges at the brain-mind–body interface (Maggio et al., 2023). Finally, our findings regarding E/I balance in children with functional seizures add to a growing literature implicating problems with energy regulation and energy flow as a mechanism underpinning functional seizures. While this study is the first to use MRS in children with FND, it builds upon prior work using EEG, fMRI, and diffusion tensor imaging to characterize and better understand the underlying neurophysiology of FND.

## Declaration of competing interest

The authors declare that they have no known competing financial interests or personal relationships that could have appeared to influence the work reported in this paper.

## Data Availability

The authors do not have permission to share data.

## Footnotes

2 mI transporter activity increases as the extracellular space is acidified and reaches as plateau at PH of 5-6 (Di Daniel et al., 2009).

3 In the resting state, Ham and colleagues (2007) found normal mI levels (panic disorder patients versus controls) in the anterior cingulate cortex and basal ganglia (Ham et al., 2007).
