# Supplementary material for: Neurometabolic alterations in children and adolescents with functional neurological disorder
Source: Neuroimage Clin. 2023 Dec 21;41:103557. doi: 10.1016/j.nicl.2023.103557 (PMC10825645; doi:10.1016/j.nicl.2023.103557)
Supplement: Supplementary data 1 [file mmc1.docx]

Supplemental Table 1: MRSinMRS

| 1. Hardware |  |
| --- | --- |
| a. Field strength [T] | 3T |
| b. Manufacturer | Siemens |
| c. Model (software version if available) | VE11C |
| d. RF coils: nuclei (transmit/receive), number of channels, type, body part | 64 channel head/neck RF coil |
| e. Additional hardware | N/A |
| 2. Acquisition |  |
| a. Pulse sequence | Point Resolved Spectroscopy and Mescher-Garwood Point Resolved Spectroscopy |
| b. Volume of Interest (VOI) locations | Anterior default mode network, posterior default mode network, supplemental motor area |
| c. Nominal VOI size [cm^3^, mm^3^] | aDMN- 25x30x35mm^3^, pDMN- 25x25x40 mm^3^, SMA-25x25x40 mm^3^ |
| d. Repetition Time (TR), Echo Time (TE) [ms, s] | TR: 2000ms, TE: 30ms |
| e. Total number of excitations or acquisitions per spectrum  In time series for kinetic studies   1. Number of Averaged spectra per time-point 2. Averaging method (e.g. block-wise or moving average) 3. Total number of spectra (acquired / in time-series) | 32 averages |
| f. Additional sequence parameters (spectral width in Hz, number of spectral points, frequency offsets)  If STEAM:, Mixing Time (TM)  If MRSI: 2D or 3D, FOV in all directions, matrix size, acceleration factors, sampling method | Spectral width=2000 Hz  For MEGAPRESS acquisition- editing pulse frequencies set at 1.9 ppm and 7.5 ppm, pulse bandwidth=70Hz |
| g. Water Suppression Method | WET |
| h. Shimming Method, reference peak, and thresholds for “acceptance of shim” chosen | Siemens advanced user 3D shimming with manual shimming if FWHM >14 Hz in the pDMN and SMA and 18 Hz in the aDMN. |
| i. Triggering or motion correction method  (respiratory, peripheral, cardiac triggering, incl. device used and delays) | N/A |
| 3. Data analysis methods and outputs |  |
| a. Analysis software | LC Model |
| b. Processing steps deviating from quoted reference or product | See “METHOD TITLE” Section |
| c. Output measure  (e.g. absolute concentration, institutional units, ratio) | Creatine ratios |
| d. Quantification references and assumptions, fitting model assumptions | Cite LC Model |
| 4. Data Quality |  |
| a. Reported variables  (SNR, Linewidth (with reference peaks)) | SNR, FWHM, CRLB |
| b. Data exclusion criteria | SNR<20, CRLB<10 for tNAA, Glx, tCho, and mI, CRLB < 20 for GABA |
| c. Quality measures of postprocessing Model fitting (e.g., CRLB, goodness of fit, SD of residual) | CRLB |
| d. Sample Spectrum |  |
